# Muscle Characteristics Comparison Analysis Reveal Differences in the Meat Quality and Nutritional Components of Three Shanghai Local Pig Breeds

**DOI:** 10.3390/foods14040569

**Published:** 2025-02-08

**Authors:** Weilong Tu, Hongyang Wang, Yingying Zhang, Wei Jiang, Chuan He, Ji Huang, Lan Bai, Yuduan Diao, Jieke Zhou, Yongsong Tan, Xiao Wu

**Affiliations:** 1 Key Laboratory of Livestock and Poultry Resources (Pig) Evaluation and Utilization, Institute of Animal Science and Veterinary Medicine, Shanghai Academy of Agricultural Sciences, Ministry of Agriculture and Rural Affairs, Shanghai 201106, China; 2 Institute of Shanghai Engineering Research Center of Breeding Pig, Shanghai 201106, China; 3 Key Laboratory of Agricultural Genetics and Breeding, Biotechnology Research Institute, Shanghai Academy of Agricultural Sciences, Shanghai Agricultural Biosecurity Evaluation and Testing Professional Technical Service Platform, Crop Ecological Environment Safety Inspection and Testing Center of the Ministry of Agriculture and Rural Affairs, Shanghai 201106, China

**Keywords:** local pig breeds, meat quality traits, amino acids, texture, flavored nucleotide

## Abstract

To study the differences in meat quality and nutritional components between the local Shanghai pig breeds Meishan pig (MS), Shawutou pig (SWT), Fengjing pig (FJ), and the commercial Duroc × Landrace × Yorkshire (DLY) crossbred pigs, and to provide data support for the selection and breeding of superior pig breeds, this study selected 30 piglets each of three local pig breeds and DLY with similar birth ages and weights around 25 kg, fed them the same daily ration with uniform nutritional components, and slaughtered ten of them at around 100 kg weight for evaluation of differences in meat quality indicators (primarily intramuscular fat content, tenderness value, texture, etc.) and amino acid content among the varieties. The results indicated significant differences among the four pig breeds in intramuscular fat content, with MS having the highest content and significant differences in tenderness value compared to the other three breeds (*p* < 0.05). In terms of texture indicators, MS and SWT differed significantly from FJ and DLY in terms of hardness and fracturability indicators (*p* < 0.05), with certain differences existing among the four breeds in other indicators. In amino acid content, the differences in total amino acid content among the three Shanghai local pig breeds were not significant (*p* > 0.05), but all were significantly higher than DLY. Further analysis revealed significant differences in amino acid content between Shanghai local pig breeds and DLY, with Shanghai local pigs showing markedly higher levels of serine, proline, isoleucine, leucine, and histidine compared to DLY (*p* < 0.05). Regarding nucleotides, the cytidine monophosphate (CMP) indicator of MS differed significantly from the other three breeds (*p* < 0.05), SWT’s uridine monophosphate (UMP) indicator differed significantly from FJ and DLY, and FJ and DLY’s inosine monophosphate (IMP) indicator was significantly higher than MS and SWT (*p* < 0.05), while SWT’s adenosine monophosphate (AMP) indicator was significantly higher than the other three breeds (*p* < 0.05). The results of this study suggest that the meat quality and nutritional composition of Shanghai local pigs are significantly superior to DLY, with MS exhibiting significantly better meat quality and nutrition compared to SWT and FJ among the three local pig breeds.

## 1. Introduction

Pork is a staple meat in China and a crucial source of animal protein in the Chinese diet, known for its tender texture, minimal connective tissue, and abundant intramuscular fat content [1]. China stands as the world’s leading pork producer, contributing to 46% of global pork production [2]. Shanghai, a prominent metropolis in China with over 24 million residents, consumes approximately 500,000 tons of pork annually. During the period from 2000 to 2010, in response to the rising national demand for meat consumption, the Duroc × Landrace × Yorkshire (DLY) crossbred pigs emerged as a primary commercial pig breed in China due to its rapid growth rate and high lean meat content, dominating a significant portion of the market [3]. With the rapid economic development in China, particularly in mega-cities such as Shanghai, there is an increasing demand for high-quality pork among the local residents, evolving from mere access to pork during the early stages of reform and opening up to a desire for premium pork products. Concurrently, in response to the escalating demand for meat products domestically, foreign pig breeds such as Duroc, Large White, and Landrace have been widely introduced alongside local pig breeds. Leveraging advantages like high lean meat content and shorter growth cycles, these foreign breeds have gradually assumed a dominant position in the Chinese domestic market [4]. However, comparative studies examining the meat quality parameters and nutritional value between Shanghai local pig breeds and imported pig breeds have been lacking. Thus, conducting a systematic comparison of meat quality and nutritional value between these two types of pig breeds is of significant importance for objectively evaluating the similarities and differences in meat quality and nutritional composition. Such studies not only hold relevance for assessing and improving local pig breeds but also offer valuable insights for the further refinement of new pig breeds in the future.

Currently, there are multiple indicators used to evaluate the quality of pork [5,6,7]. Among these, texture profile analysis (TPA) stands out as a crucial parameter for assessing meat quality, primarily conducted using a texture analyzer [8,9,10]. Widely applied in meat processing and quality control research, the texture profile analysis (TPA) serves not only for assessing the freshness of meat, evaluating the rationality of meat processing techniques, and overall meat quality, but also for enhancing meat product quality [11,12,13]. The Meishan pig (MS), the Shawutou (SWT), and the Fengjing pig (FJ) are all local breeds in Shanghai and belong to the Taihu pig series, characterized by high fertility, excellent meat quality, and robust stress resistance [14,15]. While some scholars have explored specific meat quality traits of local Chinese pig breeds [16,17,18,19,20], there has been a gap in research specifically focusing on the texture parameters, particularly comprehensive texture and flavor indicators like amino acids.

**Figure 1:**
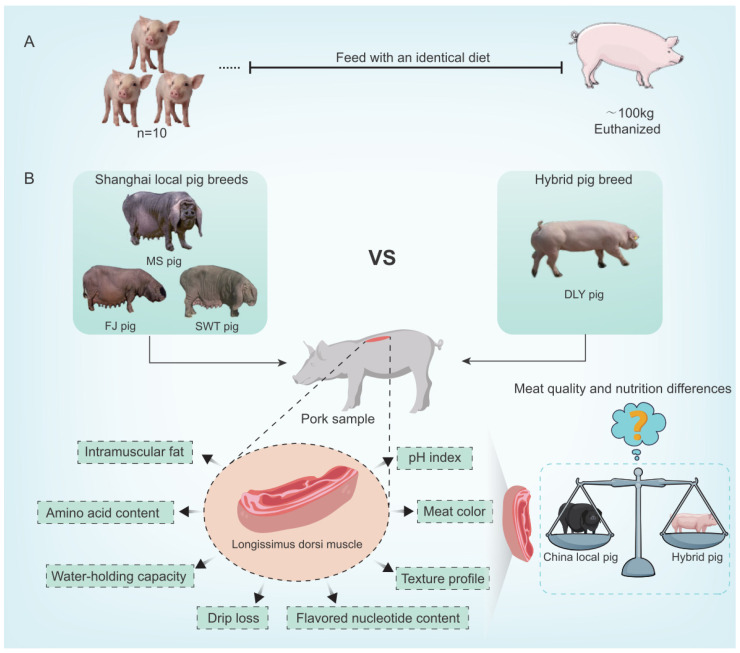
Illustration schemes and design strategies of current study. (**A**) Flowchart of the animal experiments: Ten healthy, disease-free pigs from the MS, SWT, FJ, and DLY breeds, with similar birthdates and an initial weight of approximately 25 kg, were selected for the study. All pigs were fed an identical diet with uniform nutritional content. Once the pigs reached a weight of approximately 100 kg, they were euthanized, and the *Longissimus dorsi* muscle was collected for further analysis. (**B**) Schematic diagram illustrating the evaluation and comparative analysis strategy for pork quality indicators. The *Longissimus dorsi* muscle samples from each pig breed were analyzed for various indicators, including intramuscular fat content, pH level, amino acid composition, drip loss, texture profile, water-holding capacity, meat color, and flavored nucleotide content. The values in these parameters were used to assess and compare the meat quality and nutrition difference in Shanghai local pigs and DLY crossbred pigs.

Given the current research landscape, to evaluate the meat quality of Shanghai local pigs, this study highlights the meat quality investigation of the Meishan pig (MS), the Shawutou pig (SWT), and the Fengjing pig (FJ). By examining meat quality characteristics, texture, amino acids, and comparing them with the meat quality of the Duroc × Landrace × Yorkshire (DLY) crossbred pig breed (Figure 1), this research aims to provide insights for the breeding and utilization of distinctive local pig breeds in Shanghai. Analyzing the data on pork quality assessment in the Shanghai region not only facilitates the promotion of high-quality and high-value pork products but also holds significant implications for the future direction of the industry.

## 2. Materials and Methods

### 2.1. Experimental Animals

Healthy, disease-free pigs, including MS, SWT, FJ, and DYL breeds with similar birthdates and weights, half male and half female with castrated males, were selected for the study. The MS breed was provided by the Meishan Pig Conservation Farm in Jiading, Shanghai, the SWT breed by Shanghai Shawutou Agricultural Technology Co., Ltd. (Shanghai, China), the FJ breed by Shanghai Xinnong Livestock Technology Co., Ltd. (Shanghai, China), and the DYL breed by Shanghai Yunxin Livestock Technology Co., Ltd. (Shanghai, China). The samples were refrigerated at 4 °C, and the meat quality evaluation was conducted 30–60 min later. Additionally, a portion of each muscle sample was segmented, packaged, and frozen at −20 °C for the measurement of pH at 24 h. All animal experiments were approved by the Animal Ethical Committee of the Shanghai Academy of Agricultural Sciences (No. SAASPZ0523083).

### 2.2. Sampling and Processing

Thirty nursery pigs of MS, SWT, FJ, and DYL breeds weighing around 25 kg each were fed with the same diet containing uniform nutritional components. Ten pigs per breed were slaughtered upon reaching an approximate weight of 100 kg. A single sample of the *Longissimus dorsi* muscle was collected from each pig, yielding a total of ten samples per breed for subsequent analysis. Before slaughter, the pigs were deprived of feed but allowed free access to water for 24 h. Subsequently, they were electrically stunned, exsanguinated, scalded, and rinsed. Samples were taken from the core of the *Longissimus dorsi* adjacent to the last rib immediately after exsanguination and promptly frozen in liquid nitrogen for texture index, amino acid, and nucleotide analysis. Other samples were extracted from the *Longissimus dorsi* near the third and tenth rib at 45 min post-mortem for initial meat quality trait assessments and stored at 4 °C for subsequent evaluations.

### 2.3. Meat Quality Evaluation

Comprehensive assessments of pork quality characteristics were conducted using the following methods. The pH of the *Longissimus dorsi* muscle was gauged with a pH meter with a knife blade electrode (Orion 3 star, Thermo Electron Corporation, Waltham, MA, USA), calibrated using standard buffers of pH 7.0 and 4.0 prior to conducting the measurements. Water-holding capacity was measured as drip loss as per previously established procedures with minor adjustments [21,22]. The samples of the *Longissimus dorsi* muscle measuring 2 × 3 × 5 cm were extracted, weighed, and vacuum-sealed in plastic bags. After refrigeration at 4 °C for 24 h, the samples were reweighed to calculate the drip loss based on the weight variance. The meat color was quantified using a colorimeter (SP60, X-rite, Grand Rapids, MI, USA). Samples were prepared by cutting them into cubes with dimensions of 1 cm × 1 cm × 1 cm, followed by a vertical incision. The exposed surfaces of the samples were utilized to assess lightness (L*), redness (a*), and yellowness (b*). Each measurement was conducted in triplicate. The fat content in the pork was determined using an Ankom XT10 extractor (Ankom Technology, NY, USA) to evaluate muscular texture. The tenderness of the pork was tested using a TA. XT Plus texture analyzer (TA Instruments, Newcastle, DE, USA) to assess its tenderness value by measuring elasticity and chewiness, among other factors.

### 2.4. Shear Force Analysis

Pork samples were extracted from the heat-treated *Longissimus dorsi* muscle (heating at 70 °C for 30 min) for the purpose of shear force analysis. Specifically, samples measuring 10 mm × 10 mm and approximately 50 mm in length were sectioned in alignment with the longitudinal arrangement of the muscle fibers. Shear force measurements were conducted at room temperature (approximately 20 °C) using a tenderness meter (C-LM3B, Northeast Agricultural University, Harbin, China), fitted with a V-shaped shear blade that has a 60° triangular aperture. The instrument was calibrated with the following parameters: compression mode for the test, pre-test speed of 0.2 cm/s, test speed of 0.2 cm/s, post-test speed of 0.2 cm/s, a travel distance of 0.5 cm, and an automatic triggering mechanism. The maximum peak force during the test was recorded, and the shear force data were subsequently exported as an Excel file for comprehensive chemometric analysis.

### 2.5. Texture Profile Analysis (TPA)

The texture profile analysis (TPA) of the pork samples was conducted utilizing a texture analyzer (TA Instruments, Newcastle, DE, USA). The samples were sectioned into cylindrical slices with a thickness of 10 mm, and an aluminum cylindrical probe with a diameter of 35 mm was employed for measurement. Based on preliminary investigations, the experiments were carried out at room temperature with compression ratios of 20% and 30%, and cross-head speeds of 1.0 mm/s, 2.0 mm/s, and 5.0 mm/s. This study assessed several textural parameters during the TPA, including Hardness, Fracture, Adhesion, Elasticity, Chewiness, Tackiness, Cohesive Force and Resilience. All measurements were conducted in triplicate to ensure reliability.

### 2.6. Amino Acid Composition Analysis

The amino acid analyzer efficiently separates and detects amino acids, providing accurate content data to comprehensively understand the composition of amino acids in pork. This serves as a scientific basis for assessing its nutritional value and quality characteristics. In this study, a S-433D amino acid analyzer (Sykam Technologies, Munich, Bavaria, Germany) equipped with a high-performance cation exchange column was utilized to determine the amino acid composition of pork samples. Initially, the samples underwent hydrolysis as follows: approximately 100 mg of pork sample was homogenized using a high-speed universal crusher. The resulting homogenate was subsequently transferred to a glass container, to which 10 mL of 6 mol/L hydrochloric acid (HCl) was added. After purging the mixture with nitrogen to create an inert atmosphere, it was subjected to hydrolysis at 110 °C for 22 h. Upon completion of hydrolysis, the mixture was allowed to cool and was then transferred into a 50 mL volumetric flask, where it was diluted with ultrapure water in a calibration tube. A 1 mL aliquot of the hydrolysate was extracted, and the acid was removed under reduced pressure. The remaining sample was dried, and 1 microliter of 0.02 mol/L HCl was added to ensure complete dissolution. Finally, the solution was filtered through a 0.22 µm membrane filter into an autosampler, and the analysis of amino acid composition commenced.

### 2.7. Flavored Nucleotide Analysis

The quantification of nucleotide derivatives, specifically 5′-adenosine monophosphate (AMP), 5′-inosine monophosphate (IMP), cytidine monophosphate (CMP), uridine monophosphate (UMP), and 5′-guanosine monophosphate (GMP), was conducted using high-performance liquid chromatography (HPLC) (Agilent 1260 series, Agilent Technologies, Santa Clara, CA, USA), equipped with a 4.6 × 150 mm C18 column (Agilent Technologies) and a diode array detector. Approximately 5 g of minced meat was homogenized in 25 mL of 0.7 M perchloric acid. The resulting homogenate was subjected to centrifugation at 0 °C for 15 min at 2000× *g*, followed by filtration through Whatman filter paper (No. 4). The residual pellet was re-extracted with an additional 20 mL of 0.7 M perchloric acid and subsequently filtered. The pH of the combined filtrate was adjusted to 6.5 using a 5 N KOH solution. The adjusted filtrate was then transferred to a volumetric flask and diluted to a final volume of 100 mL with 0.7 M perchloric acid. After cooling for 30 min, the solution was centrifuged at 1000× *g* for 10 min at 4 °C, and the supernatant was filtered through a 0.22-μm syringe filter. The filtered supernatant was analyzed via HPLC under the following conditions: a Nova-Pak C18 column (150 × 3.9 mm, 4 μm particles; Waters, Milford, MA, USA) was employed, with a solution using 1% trimethylamine · phosphoric acid (pH 6.5) at a flow rate of 1.0 mL/min. The injection volume was 10 μL, with a total run time of 30 min. The column temperature was maintained at 40 °C, and detection was performed at a wavelength of 254 nm.

### 2.8. Feed Nutrient Levels

In this study, pigs were fed a corn–soybean meal diet with the same formula for all four breeds. The feed formulation and nutrient contents are detailed in Table 1.

### 2.9. Statistical Analysis

The statistical analysis was performed using SPSS Statistics 22.0 software (SPSS Inc., Chicago, IL, USA), employing One-Way ANOVA for differential analysis, with significance set at *p* < 0.05, and results are presented as mean ± standard deviation (SD).

## 3. Results

### 3.1. Analysis of Meat Quality Traits

The pH values measured 24 h post-mortem of different local pig breeds and DYL are summarized in Table 2.

**Figure 2:**
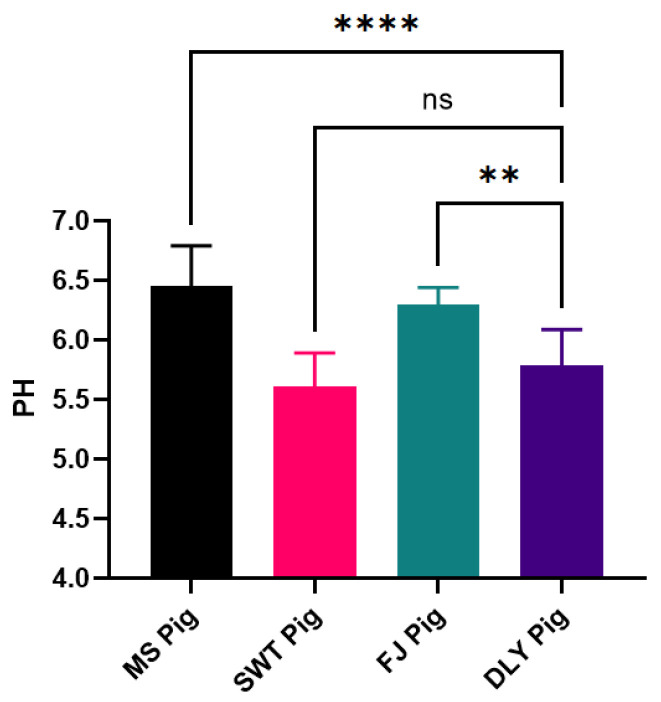
Comparison of meat pH between local Shanghai pig breeds and DYL. The values presented are the means of a minimum of three parallel tests, with error bars indicating standard deviations (SD). MS, Meishan pigs; SWT, Shawutou pigs; FJ, Fengjing pigs; DLY, Duroc × Landrace × Yorkshire (DLY) crossbred pigs. “**” means *p* < 0.01; “****” means *p* < 0.0001; ns means: Not significant. Error bars represent standard deviations calculated from at least three replicates (*n* ≥ 3).

The analysis of the pH24h index indicated that MS and FJ were significantly higher than SWT and DYL (*p* < 0.05) (Figure 2).

**Figure 3:**
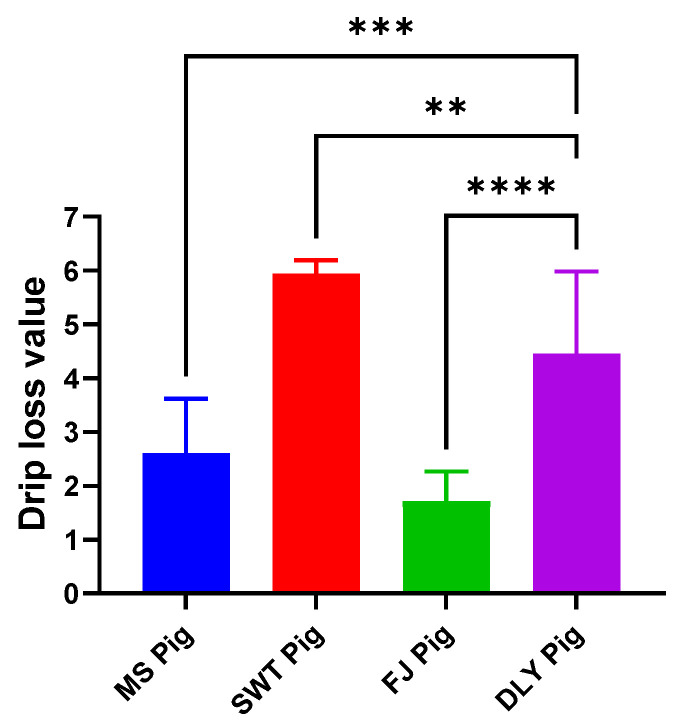
Comparison of pork drip loss between local Shanghai pig breeds and DYL. The values presented are the means of a minimum of three parallel tests, with error bars indicating standard deviations (SD). MS, Meishan pigs; SWT, Shawutou pigs; FJ, Fengjing pigs; DLY, Duroc × Landrace × Yorkshire (DLY) crossbred pigs. “**” means *p* < 0.01; “***” means *p* < 0.001; “****” means *p* < 0.0001. Error bars represent standard deviations calculated from at least three replicates (*n* ≥ 3).

Drip loss analysis revealed that MS and FJ had significantly lower drip loss compared to DYL (*p* < 0.05), with SWT showing significantly higher drip loss than DLY (*p* < 0.05) (Figure 3).

**Figure 4:**
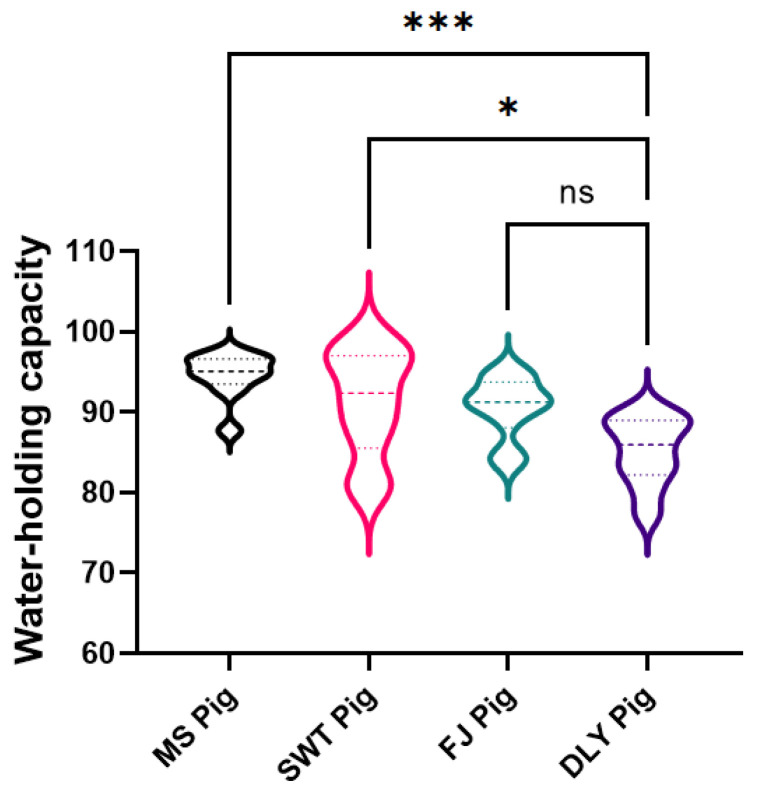
Comparison of pork water-holding capacity between local Shanghai pig breeds and DYL. The values presented are the means of a minimum of three parallel tests. MS, Meishan pigs; SWT, Shawutou pigs; FJ, Fengjing pigs; DLY, Duroc × Landrace × Yorkshire (DLY) crossbred pigs. “*” means 0.01< *p* < 0.05; “***” means *p* < 0.001; ns means: Not significant.

The shear force analysis indicated that both MS and SWT breeds exhibited significantly higher shear force than DLY (*p* < 0.05) (Figure 4); considering drip loss and shear force collectively, FJ and MS showed superior water retention abilities compared to DLY. Additionally, the meat color, which serves as the initial impression for consumers, is closely linked to consumer purchasing decisions, particularly in relation to the redness of pork [23]. We evaluated meat color using the three prevalent color parameters: Lightness (L*), Redness (a*), and Yellowness (b*).

**Figure 5:**
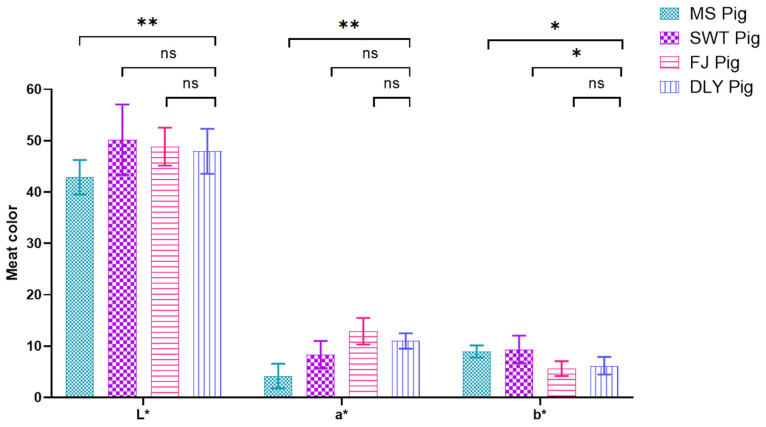
Comparison of meat color between Shanghai local pig breeds and DYL. The values presented are the means of a minimum of three parallel tests, with error bars indicating standard deviations (SD). L*, Lightness; a*, redness; b*, yellowness. MS, Meishan pigs; SWT, Shawutou pigs; FJ, Fengjing pigs; DLY, Duroc × Landrace × Yorkshire (DLY) crossbred pigs. “*” means 0.01 < *p* < 0.05; “**” means *p* < 0.01; ns means: Not significant. Error bars represent standard deviations calculated from at least three replicates (*n* ≥ 3).

In this study, significant differences were observed among the breeds for the L* value (*p* < 0.05), with DYL showing significantly higher a* value than MS and SWT exhibiting significantly higher b* value than FJ and DYL (Figure 5).

**Figure 6:**
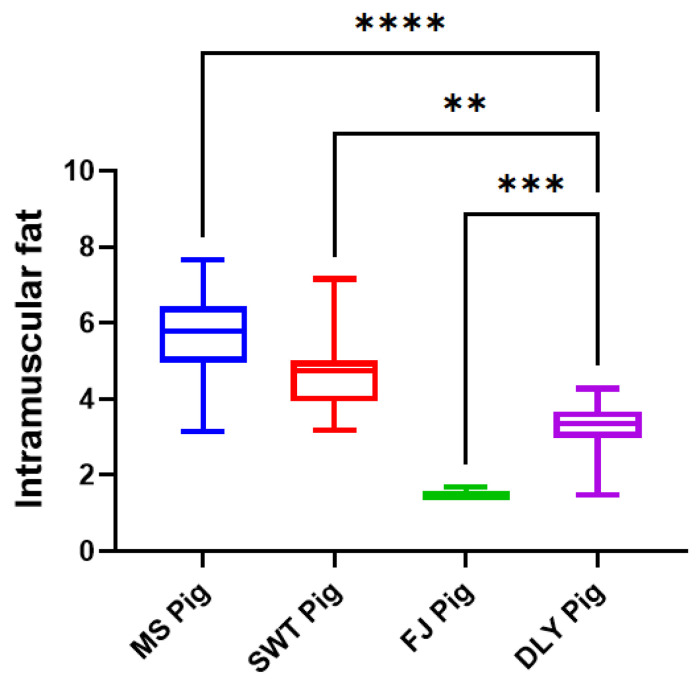
Comparison of meat intramuscular fat between local Shanghai pig breeds and DYL. The values presented are the means of a minimum of three parallel tests, with error bars indicating standard deviations (SD). MS, Meishan pigs; SWT, Shawutou pigs; FJ, Fengjing pigs; DLY, Duroc × Landrace × Yorkshire (DLY) crossbred pigs. “**” means *p* < 0.01; “***” means *p* < 0.001; “****” means *p* < 0.0001. Error bars represent standard deviations calculated from at least three replicates (*n* ≥ 3).

Regarding fat content, MS had the highest intramuscular fat content, significantly higher than the other three breeds (*p* < 0.05), with significant variations observed across all four breeds (*p* < 0.05) (Figure 6).

**Figure 7:**
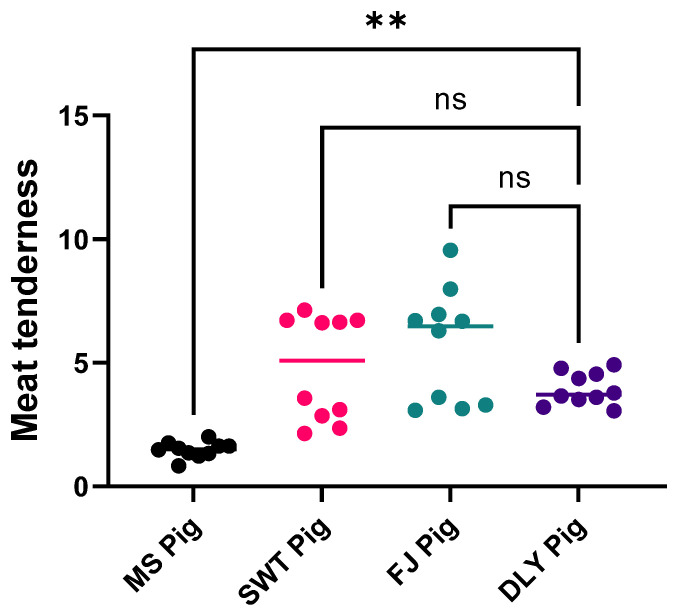
Comparison of meat tenderness between local Shanghai pig breeds and DYL. The values presented are the means of a minimum of three parallel tests. MS, Meishan pigs; SWT, Shawutou pigs; FJ, Fengjing pigs; DLY, Duroc × Landrace × Yorkshire (DLY) crossbred pigs. “**” means *p* < 0.01; ns means: Not significant.

Moreover, MS exhibited the lowest tenderness value, significantly lower than SWT and FJ (*p* < 0.05), indicating that MS pork required the least shear force (Figure 7).

### 3.2. Texture Profile Analysis

Meat texture, encompassing hardness, fracturability, adhesiveness, elasticity, chewiness, gumminess, cohesiveness, and springiness, is a critical parameter for assessing meat quality. Hardness, a key factor determining meat quality, directly reflects the texture and affects chewiness and cohesiveness in texture profile analysis. Fracturability assesses the texture’s surface response to reveal the sample’s perception. Adhesiveness refers to the negative forces produced after sample compression. Elasticity measures the sample’s recovery height between bites. Chewiness quantifies the force or energy required to chew the sample. Gumminess evaluates the semi-solid texture of the sample. Cohesiveness compares the area ratio between the first and second compressions, while springiness measures the sample’s speed and toughness in recovering from deformation.

The textural analysis results of the three Shanghai local pig breeds and DYL are presented in Table 3. The findings indicated that the hardness of FJ pork was significantly lower than that of MS and SWT (*p* < 0.05). Fracturability in MS and SWT was significantly higher than in FJ and DYL (*p* < 0.05), with FJ also showing higher values than DYL (*p* < 0.05). Adhesiveness in MS pork was significantly lower than in SWT and DYL (*p* < 0.05). Moreover, FJ pork exhibited significantly higher elasticity than the other three breeds. No significant differences were observed in gumminess, cohesiveness, and springiness among the four breeds (*p* > 0.05).

### 3.3. Amino Acid Content Analysis

Amino acids are vital nutrients and flavor compounds in pork, significantly impacting its quality. The results of amino acid content analysis in the three Shanghai local pig breeds and DYL are presented in Table 4. The total amino acid content did not differ significantly among the local pig breeds (*p* > 0.05), but all were significantly higher compared to DLY (*p* < 0.05). While no significant differences were observed among the breeds in aspartic acid, threonine, and arginine content (*p* > 0.05), SWT exhibited significantly higher levels of glutamic acid, alanine, and norleucine compared to FJ and DYL (*p* < 0.05). Serine content in SWT was significantly higher than in FJ and DYL (*p* < 0.05), and MS demonstrated significantly higher serine content than in FJ and DYL (*p* < 0.05). Methionine and tryptophan content were significantly higher in MS and SWT compared to FJ and DYL (*p* < 0.05), and MS had significantly higher levels of tyrosine and phenylalanine compared to the other three breeds (*p* < 0.05).

### 3.4. Determination of Flavored Nucleotide Content

Nucleotides are essential nutrients for maintaining normal immune function in the body, enhancing immunity, and the ability to resist infections. Additionally, nucleotides play a crucial role in the sensory perception of meat, as they enhance the umami taste by interacting with other umami components, serving as important indicators of meat quality. The results of flavored nucleotide content determination in the three Shanghai local pig breeds and DYL pork are presented in Table 5. The findings demonstrate that CMP in MS pork was significantly higher than in the other three breeds (*p* < 0.05), while UMP, IMP in FJ pork, and DYL were significantly higher than in SWT and MS (*p* < 0.05). Furthermore, AMP in SWT pork was significantly higher than in the other three breeds (*p* < 0.05), while GMP did not exhibit significant differences across the four breeds (*p* > 0.05).

## 4. Discussion

Shanghai has a long history of pig breeding, leading to the development of distinctive pig breeds such as Meishan pig (MS), Fengjing pig (FJ), and Shawutou pig (SWT) through extensive natural selection. The meat of these pigs is characterized by its vibrant red color, tender texture, and rich yet non-greasy taste, fulfilling the consumption demand of local residents in Shanghai for pork. Among these breeds, the Meishan (MS) pig holds the reputation of being the most prolific pork producer globally and is hailed as a “national treasure” [24,25]. The commercial Duroc × Landrace × Yorkshire (DLY) crossbred pig is a novel hybrid pig breed created from the crossbreeding of three varieties of boars: Landrace, Yorkshire, and Duroc [26,27,28]. In comparison with native Chinese pig breeds, the DLY exhibits advantages in faster weight gain, higher feed conversion efficiency, and relatively higher lean meat content [4,27,28]. Currently, there is a lack of comparative data on the meat quality and nutritional components between the local Shanghai pig breeds and the DLY in China. To address the disparities in meat quality and nutritional attributes among the indigenous Shanghai pig breeds Meishan pig (MS), Fengjing pig (FJ), Shawutou pig (SWT), and the Duroc × Landrace × Yorkshire (DLY) crossbred pig, and to offer empirical data to facilitate the identification and breeding of superior pig breeds, the present study was undertaken.

Quality characteristics of pork include various indicators such as pH value, drip loss, water holding capacity, meat color, intramuscular fat, and tenderness, all of which are key factors in assessing pork quality [5,29,30,31]. Due to the acidification process in pork, the pH value exhibits a pattern of initial decrease followed by an increase post-slaughter [32]. In this study, the pH value was measured at 45 min and 24 h post-mortem; however, due to biosafety concerns, the pH values were uniformly assessed at 24 h. The results revealed that the pH values of MS and FJ were significantly higher than DLY (*p* < 0.05), while the pH value of SWT was slightly lower. This discrepancy in SWT may be attributed to prolonged post-slaughter meat aging, leading to incomplete acidification. The moisture content in muscles exists in three main forms: bound water, immobilized water, and free water [33]. Drip loss, a significant parameter in meat quality evaluation, occurs during slaughter, storage, processing, and transportation processes [34,35,36]. Meat possesses inherent mechanisms, both physical and chemical, to retain moisture. Water holding capacity serves as a crucial indicator of meat quality [37,38]; water is stored in different spatial structures within muscles, including within myofibers, between myofibers, between myofibers and cell membranes, intercellularly, and between muscle bundles. Drip loss and water holding capacity are essential components of meat quality evaluation and show a negative correlation [39], a relationship confirmed by the results of this study.

Meat color, comprising L* (brightness), a* (redness-greenness), and b* (yellowness-blueness), undergoes changes during the acidification process, impacting the proportions of muscle fibers and myoglobin concentrations [40,41]. In our study, MS and SWT exhibited slightly superior meat color compared to DLY, with FJ displaying less desirable color characteristics. Consumers generally prefer lean meat but favor pork with higher intramuscular fat content, as regular consumption of such meat can help prevent atherosclerosis and diabetes [42,43]. In this study, both MS and SWT showed significantly higher intramuscular fat content than DLY, with MS having the highest content [44]. On the other hand, FJ displayed lower intramuscular fat content, potentially linked to slaughter weight. Tenderness is a crucial factor determining consumer satisfaction and purchase intent towards pork [45]. Various methods exist to evaluate tenderness, with shear force analysis being widely accepted [46,47,48]. The current study found that MS exhibited significantly lower tenderness compared to the other two local pig breeds (*p* < 0.05) and DLY, albeit without statistical significance (*p* > 0.05).

Texture analysis includes various parameters such as hardness, fracturability, adhesiveness, elasticity, chewiness, cohesiveness, gumminess, and springiness [47,49,50]. In our study, MS demonstrated significantly higher hardness and fractur ability compared to the other three breeds, while adhesiveness was significantly lower. The overall texture attributes of DLY were comparable to those of the local pig breeds. The relationship between hardness, chewiness, and adhesiveness typically shows a negative correlation and positively correlates with tenderness [51]. However, our results exhibited a distinct trend where the interrelation was not observed, likely due to the unique properties of the pork samples. Overall, MS displayed significantly higher hardness and fracturability, lower adhesiveness, and no substantial difference in comparison to the other three breeds.

Pork is rich in protein, with its protein content determining the amino acid levels. Amino acids are crucial nutritional and flavor components in pork and have a significant impact on pork quality [52]. Amino acids are categorized into essential and non-essential types, with humans requiring eight essential amino acids from food sources: lysine, tryptophan, phenylalanine, methionine, threonine, isoleucine, leucine, valine, and histidine. Among the reported amino acids, glutamic acid, and aspartic acid are directly linked to the umami taste of pork, representing key umami amino acids [53]. Additionally, alanine and glycine have been reported as essential amino acids influencing pork flavor [54]. The metabolism of different amino acids in animal bodies can affect pork texture, with cysteine serving as a precursor to methionine, a vital amino acid influencing pork drip loss [55]. Amino acids also impact the health status of pigs, subsequently affecting pork quality; for instance, the addition of glutamine can enhance a pig’s immunity [56], leucine can regulate pork muscle fat composition and lipid metabolism-related gene expression, and influence pig growth rate, thus improving pork quality [57]. In this study, the total amino acid content did not significantly differ among the three local pig breeds (*p* > 0.05), but all were significantly higher than DLY (*p* < 0.05), indicating superior amino acid content in local pig pork compared to DLY, supporting findings from previous studies [58,59]. Conversely, our results diverge from earlier findings concerning five pig breeds in China (manor black pigs, Tibetan black pigs, Pipa pigs, Yihao native pigs, and white pigs), where significant variations in amino acid contents were observed among these breeds. Notably, the Tibetan black pigs had the highest total amino acid content compared to the other breeds [20]. In contrast, the three Shanghai local pig breeds in our study showed minimal differences in amino acid content, potentially attributed to their long-term adaptation to local environmental and climatic conditions, which may have influenced the selection for desirable traits in these breeds. Additionally, aspartic acid, a key flavor-enhancing amino acid, was significantly higher in the pork of all three Shanghai local pig breeds compared to DLY, suggesting superior flavor in the pork of the three Shanghai local pig breeds, a preference corroborated by the popularity of market black pigs among consumers.

There are five nucleotides, including CMP, UMP, AMP, GMP, and IMP, each with distinct functions and collectively essential nucleotides in the human body, as nucleic acids are constituted of nucleotides [60]. Nucleic acids are indispensable components for all known forms of life, encompassing deoxyribonucleic acid (DNA) and ribonucleic acid (RNA). Nucleotides are common nutritional components in human diets, especially crucial when endogenous nucleotide synthesis is disrupted due to certain diseases, emphasizing the importance of nucleotide intake through animal products [61]. Nucleotides are vital nutritional components for maintaining normal immune function in organisms, promoting immune response and infection resistance with adequate intake, while also fostering growth, development, and repair of intestinal cells [62,63,64]. Moreover, nucleotides (CMP, UMP, AMP, GMP, and IMP) play a significant role in meat sensory attributes, enhancing meat umami when combined with other flavor components, and serving as a pivotal indicator in assessing meat quality [65,66]. In the study, conducted by Zhang et al., an analysis of nucleotide concentrations across five different pork breeds revealed that inosine monophosphate (IMP) was the predominant nucleotide present [20]. Notably, the IMP content in Yihao native pigs was statistically higher (*p* < 0.05) than that in white pigs. Furthermore, white pigs exhibited a significantly elevated IMP level compared to Tibetan black pigs, which in turn had a higher IMP concentration than both Manor black pigs and Pipa pigs, with Pipa pigs showing the lowest levels. Conversely, our research demonstrated that the cytidine monophosphate (CMP) levels in the MS breed were significantly greater than those found in the other three breeds. In addition, uridine monophosphate (UMP) and IMP levels were markedly higher in the FJ breed compared to those in MS and SWT. Interestingly, adenosine monophosphate (AMP) content in SWT was significantly greater than in the other three breeds (*p* < 0.05). These findings underscore a distinctive pattern in nucleotide profiles, emphasizing the nutritional advantages and metabolic potential of local pig breeds compared to DLY pigs in our study. This variation in nucleotide content may be attributed to differences in genetic backgrounds, dietary factors, and environmental conditions influencing the metabolomic pathways of these breeds. Understanding these differences can provide insights into the breeding and management practices that could enhance the quality of pork products.

## 5. Conclusions

This study aimed to investigate the differences in meat quality and nutritional components among Shanghai local pig breeds Meishan pig (MS), Fengjing pig (FJ), Shazhu pig (SWT), and Duroc × Landrace × Yorkshire (DLY) crossbred pig. The results demonstrated significant variations in intramuscular fat content among the four pig breeds, with MS exhibiting the highest intramuscular fat content and significantly different tenderness values compared to the other three breeds. Regarding texture attributes, MS and SWT showed significant differences in hardness and fracture toughness compared to FJ and DLY, along with variations in other texture indicators. In terms of amino acid content, the total amino acid content did not significantly differ among the three Shanghai local pig breeds but was significantly higher than DLY. Further analysis revealed that Shanghai local pigs exhibited significantly higher levels of serine, alanine, isoleucine, leucine, and histidine compared to DLY.

**Figure 8:**
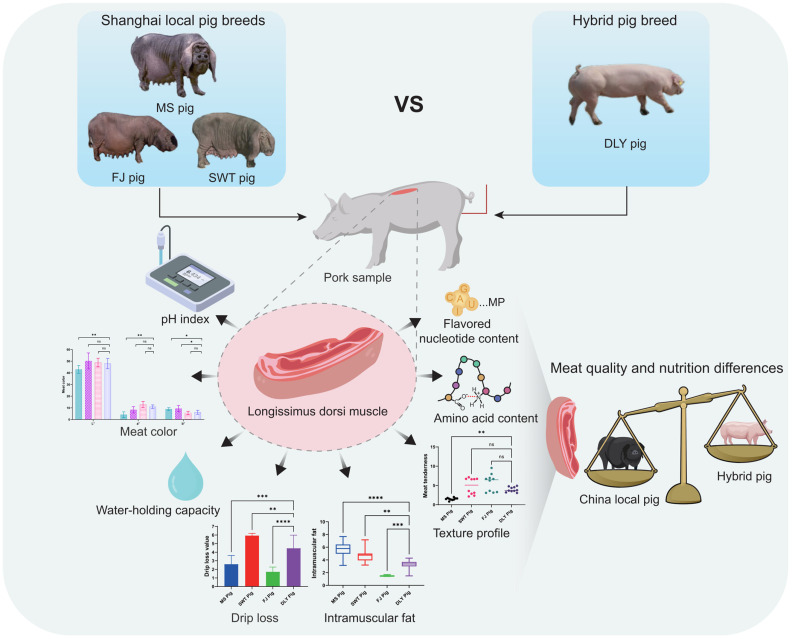
Schematic diagram demonstrating that the meat quality and nutritional components of Shanghai local pigs exceed those of DLY crossbred pigs. “*” means 0.01 < *p* < 0.05; “**” means *p* < 0.01; “***” means *p* < 0.001; “****” means *p* < 0.0001; ns means: Not significant. Error bars represent standard deviations calculated from at least three replicates (*n* ≥ 3).

These results collectively indicate that the meat quality and nutritional components of Shanghai local pigs surpass those of DLY, with MS exhibiting notably superior meat quality and nutrition compared to SWT and FJ within the local pig breeds (Figure 8). These findings offer significant scientific insights into the differences in meat quality and nutritional components among various pig breeds.

## Figures and Tables

**Table 1:** Ingredient composition and nutrient composition of a pig diet.

Ingredient	Content (%)	Nutrient Composition	
Corn	65	Metabolic energy (MJ/kg)	13.9
Soybean meal	18	Crude protein (%)	18.0
Wheat bran	12	Total phosphorus (%)	0.49
Limestone	1.2	Lysine (%)	0.85
Dicalcium phosphate	0.86	Methionine + cysteine (%)	0.78
Salt	0.30	Threonine (%)	0.56
Phytase	0.01	Tryptophan (%)	0.20
L-Lysine hydrochloride	0.52		
DL-Methionine	0.17		
Threonine	0.17		
Premix	1		

**Table 2:** Comparison of meat quality traits between Shanghai local pig breeds and DYL.

Indicator Parameters		Pig Breeds			
		MS	SWT	FJ	DYL
pH _24h_		6.46 ± 0.34 **	5.61 ± 0.29	6.3 ± 0.14 **	5.78 ± 0.3
Drip loss (%)		2.61 ± 1.01 **	5.93 ± 0.26 *	1.72 ± 0.55 **	4.76 ± 1.73
Water Holding Capacity		94.61 ± 2.86 **	91.25 ± 6.73 *	90.6 ± 3.83	85.21 ± 4.28
Meat color	Lightness (L)	42.91 ± 3.35 **	50.21 ± 6.87 **	48.85 ± 3.50 *	47.96 ± 4.16
	Redness (a)	4.18 ± 2.41 **	8.35 ± 2.64	12.9 ± 2.59	11 ± 1.51
	Yellowness (b)	8.97 ± 1.18 *	9.37 ± 2.67 *	5.63 ± 1.45	6.2 ± 1.71
Intramuscular fat (%)		5.66 ± 1.24 **	4.74 ± 1.06 *	1.49 ± 0.08 **	3.22 ± 0.77
Tenderness		1.48 ± 0.47 **	4.79 ± 2.13	5.73 ± 2.3	3.35 ± 0.74

Note: The numbers in each column represent the value of the corresponding indicator parameter in each row. “*” means 0.01 < *p* < 0.05; “**” means *p* < 0.01.

**Table 3:** Comparison of pork texture parameters between local Shanghai pig breeds and DYL.

Indicator Parameters	Pig Breeds			
	MS	SWT	FJ	DYL
Hardness	7624.86 ± 949.42	7811.35 ± 2131.51	6015.34 ± 289.66	7126.38 ± 358.97
Fracture	7526.55 ± 961.44 **	7625.05 ± 1759.39 **	6321.95 ± 452.37 *	4632.59 ± 386.54
Adhesion	205.87 ± 140.55 **	328.64 ± 137.92	314.81 ± 48.69	344.71 ± 68.39
Elasticity	0.76 ± 0.04 *	0.77 ± 0.13	0.99 ± 0.07 **	0.69 ± 0.06
Chewiness	3322.12 ± 681.16 **	4297.98 ± 2714.84	2854.37 ± 198.66 **	4156.37 ± 298.64
Tackiness	4403.37 ± 826.37	5475.10 ± 2502.97 *	4445.83 ± 392.27	4098.08 ± 371.09
Cohesive force	0.58 ± 0.09 *	0.71 ± 0.30	0.65 ± 0.05	0.66 ± 0.06
Resilience	0.08 ± 0.01	0.11 ± 0.01	0.11 ± 0.02	0.08 ± 0.02

Note: The numbers in each column represent the value of the corresponding pork texture parameters in each row. “*” means 0.01 < *p* < 0.05; “**” means *p* < 0.01.

**Table 4:** Comparison of pork amino acid content between local Shanghai pig breeds and DYL.

Amino Acid Types	Pig Breeds			
	MS	SWT	FJ	DYL
Aspartate	2.36 ± 0.30	1.87 ± 0.11	2.29 ± 0.04	1.66 ± 0.14
Glutamate	3.10 ± 0.49 *	3.21 ± 0.18 **	2.96 ± 0.53 *	2.72 ± 0.20
Serine	0.83 ± 0.16 **	0.73 ± 0.05 *	0.66 ± 0.10	0.63 ± 0.09
Threonine	0.95 ± 0.33	0.87 ± 0.05	0.88 ± 0.12	0.8 ± 0.07
Alanine	1.12 ± 0.08 *	1.19 ± 0.07 **	0.98 ± 0.26	0.99 ± 0.09
Valine	1.02 ± 0.07 *	1.09 ± 0.07 **	0.90 ± 0.21	0.91 ± 0.07
Methionine	0.47 ± 0.17 *	0.56 ± 0.03 **	0.49 ± 0.11 *	0.38 ± 0.05
Isoleucine	0.93 ± 0.07 *	0.97 ± 0.05 *	0.82 ± 0.17	0.85 ± 0.07
Leucine	1.63 ± 0.12 **	1.64 ± 0.10 **	1.43 ± 0.30	1.42 ± 0.12
Tyrosine	0.83 ± 0.06 **	0.63 ± 0.10 *	0.66 ± 0.06 *	0.60 ± 0.06
Phenylalanine	0.84 ± 0.06 **	0.63 ± 0.20 *	0.68 ± 011	0.70 ± 0.07
Histidine	0.73 ± 0.14 *	0.98 ± 0.07 **	0.81 ± 0.20	0.83 ± 0.09
Lysine	1.74 ± 0.12	1.80 ± 0.11 **	1.53 ± 0.34	1.49 ± 0.14
Arginine	1.28 ± 0.10	1.27 ± 0.08	1.40 ± 0.82	1.12 ± 0.21
Total	19.05 ± 1.95 **	18.77 ± 1.10 **	17.64 ± 3.55 **	16.58 ± 1.49

Note: The numbers in each column represent the value of the corresponding pork amino acid in each row. “*” means 0.01 < *p* < 0.05; “**” means *p* < 0.01.

**Table 5:** Comparison of pork flavored nucleotide content between local Shanghai pig breeds and DYL.

Amino Acid Types	Pig Breeds			
	MS	SWT	FJ	DYL
CMP	45.47 ± 6.78 **	26.36 ± 10.93	27.15 ± 5.48	23.51 ± 3.87
UMP	16.95 ± 4.10	22.27 ± 9.45	42.71 ± 13.02 **	40.84 ± 1.89 **
GMP	65.34 ± 4.02	70.01 ± 45.38	45.97 ± 11.20	68.29 ± 17.86
IMP	1.28 ± 0.29	0.67 ± 0.23	2.10 ± 0.86	2.14 ± 0.19 **
AMP	66.38 ± 8.46	196.50 ± 50.93 **	51.86 ± 16.19	63.92 ± 2.11

Note: The numbers in each column represent the value of the corresponding pork flavored nucleotide content in each row. “**” means *p* < 0.01.

## Data Availability

The original contributions presented in the study are included in the article, further inquiries can be directed to the corresponding authors.
